# A New Furostanol Glycoside from *Tribulus terrestris*

**DOI:** 10.3390/molecules15020613

**Published:** 2010-01-27

**Authors:** Yajuan Xu, Yonghong Liu, Tunhai Xu, Shengxu Xie, Yunshan Si, Yue Liu, Haiou Zhou, Tonghua Liu, Dongming Xu

**Affiliations:** 1 Academy of Traditional Chinese Medicine of Jilin Province, Changchun 130021, China; E-Mail: xyj6492@sohu.com (Y.X.); 2 Department of Traditional Chinese Medicine Chemistry, School of Traditional Chinese Medicine, Beijing University of Chinese Medicine, Beijing 100102, China; 3 Tianjin University of Traditional Chinese Medicine, Tianjin 300193, China; 4 Key Laboratory of Marine Bio-resources Sustainable Utilization, South China Sea Institute of Oceanology, Chinese Academy of Sciences, No. 164 West Xingang Road, Guangzhou 510301, China; E-Mail: yonghongliu@scsio.ac.cn

**Keywords:** *Tribulus terrestris* L., zygophyllaceae, furostanol glycoside

## Abstract

Besides two known glycosides, a new furostanol glycoside was isolated from the Fruits of *Tribulus terrestris* L. The structure of the new furostanol glycoside was established as 26-*O*-β-D-glucopyranosyl-(25*S*)-5α-furostane-20(22)-en-12-one-3*β*, 26-diol-3-*O*-*α*-L-rhamnopyranosyl-(1→2)-[*β*-D-glucopyranosyl-(1→4)]-*β*-D-galactopyranoside (**1**) on the basis of 1D and 2D-NMR techniques, including COSY, HMBC, and HMQC correlations.

## 1. Introduction

*Tribulus terrestris* L. is an annual plant found around the world. Its fruits have been used in traditional Chinese medicine for treatment of eye problems, edema, abdominal distention, emission, morbid leucorrhea, sexual dysfunction and veiling. It also has been used as a medicine in India, South Africa and Japan. Some steroidal saponins have previously been isolated from this plant. Many pharmaceutical preparations and food supplements with these saponins as the active compound have been commercially available. Examples of these are “Tribestane” and “Vitanone”, which have been used to treat impotency, as well as “tribusaponins” and “Xin-nao-shu-tong”, which have been used for the treatment of cardiovascular disease [1,2]. Recently, more than fifty steroidal saponins have been isolated from this plant [3,4,5,6,7,8,9,10,11,12,13]. In a preceding paper, we had reported the isolation and structural elucidation of three steroidal glycosides obtained from the fruits of this plant [14,15]. As a continuation to this study, we now describe the isolation and structural elucidation of a new furostanol glycoside obtained from the crude saponins of the fruits of *T. terrestris*.

Our investigation on the constituents in the ethanol extract of the plant led to the isolation of a new furostanol glycoside **1**, along with two known constituents: chloromaloside A (**2**) [16] and 25(*R, S*)-5α-spirostane-12-one-3-β-ol-3-*O*-*β*-D-xylopyranosyl(1→2)-[*β*-D-xylopyranosyl(1→3)]-glucopyranosyl-(1→4)-[*α*-L-rhamnopyrano-syl(1→2)]-*β*-D-galactopyranoside (**3**) [17]. Herein, we describe the isolation and structure elucidation of the new compound **1**.

## 2. Results and Discussion

Compound **1**, isolated as a white powder, was deduced to possess a furostanol structure by the Ehrlich test. Its molecular formula was determined as C_51_H_82_O_23_ on the negative ion HRESIMS ([M-H]^−^, *m/z* 1061.5160). In the positive- and negative-ion ESI-MS of **1** quasimolecular ion peaks were observed at *m/z* 1063 [M+H]^+^ and *m/z* 1061 [M-H]^−^, respectively. Furthermore, fragment ion peaks at *m/z* 917 [M+H-146]^+^, 755 [M+H-162-146]^+^, 593 [M+H-162-146-162]^+^, 431 [M+H-162-146-162-162]^+^ and 899 [M-H-162]^−^, 753 [M-H-162-146]^−^ were observed in the positive- and negative-ion ESIMS of **1,** respectively. The ^1^H-NMR spectrum of **1** showed diagnostic signals of four methyl groups at δ 0.66 (3H, s, H_3_-18), 0.78 (3, s, H_3_-19), 1.62 (3H, s, H_3_-21), 0.90 (3H, d, *J* = 6.8 Hz, H_3_-27), and signals of two oxymethines at δ 3.81 (1H, m, H-3), 4.43 (1H, m, H-16) and one oxymethylene at *δ* 3.48 (1H, dd, *J* = 7.5, 9.5 Hz, H_a_-26), 4.07 (1H, m, H_b_-26), and four anomeric proton doublets at *δ* 4.79 (1H, d, *J* = 7.3 Hz, gal H-1), 5.06 (1H, d, *J* = 7.6 Hz, glc H-1), 6.10 (1H, s, rha H-1), 4.70 (1H, d, *J* =7.6 Hz, glc′-H-1′). The ^13^C-NMR specrum of **1** showed signals of four angular methyl groups (*δ* 12.0, 11.8, 14.3, and 17.2), and four anomeric carbons (δ 100.1, 102.5, 105.3, and 107.3). The assignments of the aglycone moiety were determined by DEPT, HMQC, HMBC, and comparison with the aglycone moiety of tribufuroside C [15]. The ^1^H- and ^13^C-NMR signals of the aglycone moiety of **1** were superimposable on those of tribufuroside C, indicating the aglycone of **1** was same as that of tribufuroside C, which is 5*α*-furost-20(22)-en-12-one-3*β*, 26-triol. An acidic hydrolysis of **1** with mineral acid afforded galactose, glucose, and rhamnose. In the HMBC experiment of **1**, long-range correlations were observed between the following protons and carbons: H-1 of gal at *δ* 4.79 and C-3 of the aglycone at *δ* 76.8; H-1 of glc at *δ* 5.06 and C-4 of gal at *δ* 81.4; H-1of rha at *δ* 6.10 and C-2 of gal at δ 77.1, and H-1′ of glc’ at *δ* 4.70 and C-26 of the aglycone at *δ* 75.4. On the basis of all of this evidence, **1** was identified as 26-*O*-*β*-D-glucopyranosyl-(25*S*)-5α-furostane-20(22)-en-12-one-3*β*, 26- diol -3-*O*-α-L-rhamnopyranosyl-(1→2)-[*β*-D- glucopyranosyl-(1→4)]-*β*-D-galactopyranoside.

**Figure 1:**
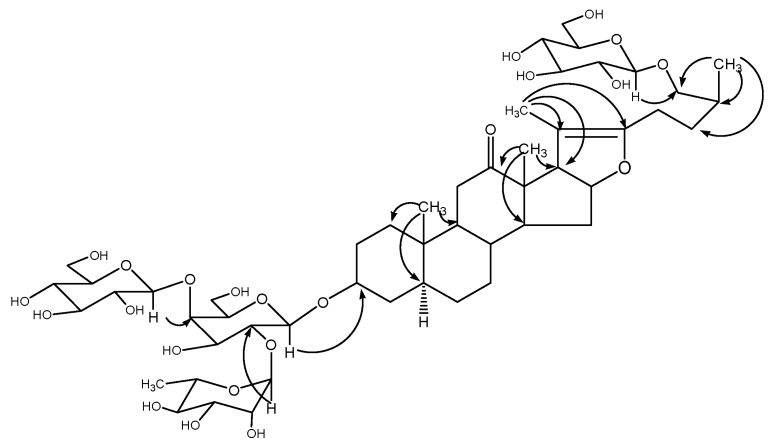
Structure and key HMBC correlations (H → C) compound **1**.

## 3. Experimental 

### 3.1. General

The optical rotations were measured on a WZZ-15 autopolarimeter. The HRESIMS was recorded on IonSpec HiResESI FT-ICR and the ESIMS was recorded on LCQ-1700 ESIMS instrument. The NMR spectra were obtained on a Bruker AM-500 instrument, using TMS as internal standard. HPLC was carried out using a Waters 600 HPLC system (pump: waters 600 E power, a DDL-31 light scattering Detector detected at 128 C and a waters 486 UV Detector detected at 203nm) equipped with a Waters Novapak C_18_ column (i.d. 25 × 100 mm and 8 × 100 mm) with a mobile phase flow rate of 10 mL min^−1^ for prep HPLC and 1 mL min^−1^ mobile phase for analysis, respectively. Column chromatography was carried out on silica gel (200-300 mesh Qingdao Marine Chemical Inc., P. R. China), and macroporous resin D_101_ made by Tianjin Gel Co. (Tianjin, China). Reversed phase column chromatographic separations were carried out using ODS (200-300 mesh) provided by Fuji Chem. TLC was performed on silica gel plates (Kieselgel 60 F254, Merck) and RP C_18_ silica gel plates (Merck). The spots on TLC were visualized by UV light (254/366 nm) and sprayed with 10% H_2_SO4, followed by heating.

### 3.2. Plant Material

The fruits of *Tribulus terrestris* L. were purchased from Jilin Medicinal Material Corporation, Changchun, China and identified by Professor Minglu Deng, Changchun College of Traditional Chinese Medicine. A voucher specimen (No.06091) has been deposited in the Herbarium of Academy of Traditional Chinese Medicine and Material Medica of Jilin Province.

### 3.3. Extraction and Isolation

The dry fruits of *T. terrestris* L*.* (7.5 kg) were crushed and extracted with 60% EtOH (70–80 °C, 3 × 25 L). The 60% EtOH solution were heated on steam bath to remove EtOH. The water solution was chromatographed on 1.0 kg D_101_ porous resin, eluting with water until the elute was colorless and then with 70% EtOH (6 L). The EtOH solution was further subjected to neutral resin to remove most of color material and then evaporated to dryness to give crude saponins (12 g). Part of the crude saponins (12 g) was chromatographed on silica gel (800 g, 200 mesh) with CHCl_3_-MeOH-H_2_O gradients 1:0:0, 50:10:1 to 10:10:1 and finally with MeOH, 250 mL per fraction, monitoring on TLC (CHCl_3_-MeOH-H_2_O-n-BuOH = 60:40:45:6), to give Fr.1 to Fr.7. Fr.4 (20:10:1, Part 37-48, 3.0g) was submitted to repeated column chromatography on silica gel (200 mesh, 310 g), eluted with CHCl_3_-MeOH-n-BuOH (250ml per part, TCL / CHCl_3_-MeOH-*n*-BuOH = 8:2:1) to afford Fr.4-1 to Fr.4-6. Fr.4-2 (part 9-12, 120 mg) was dissolved in MeOH to give compound **1** (31 mg) as a white amorphous powder. Fr.4-4 (part 20-24, 200 mg) was subjected to HPLC eluting with 60%, 50%, 45% MeOH in turn to give compound **2** (45% MeOH, Rt = 18 min, 36 mg). Fr.5 (10:10:1 part 32-43, 1.1 g) was subjected to HPLC eluting with 50%, 45% MeOH to give compound **3** (45% MeOH, Rt = 16 min, 46 mg).

### 3.4. Acid Hydrolysis of ***1***

Compound **1** (10 mg) were dissolved in 1 mol/L HCl in MeOH-H_2_O (1:1) and refluxed for 2 h. The reaction mixture was neutralized with NaHCO_3_. The water phase was chromatographed on the silica gel HPTLC with the system of *n*-BuOH-i-PrOH-H_2_O (10:5:4, homogenous), then the brown coloured spots were visualized by spraying with phenylamine-*ortho*-benzenedicarboxylic acid reagent followed by heating. The glucose, galactose and rhamnose were detected by comparison with the authentic samples.

### 3.5. Characterization of Compound ***1***

White amorphous powder, mp 228 °C (dec.); [α] _D_^25^ –63.6° (c 0.25; MeOH); HRESIMS *m/z*: found 1061.5160 [M-H]^−^ (calcd. for C_51_H_82_O_23_-H, 1061.5168); ESI MS *m/z*: 1063 [M+H]^+^, 917 [M+H-146]^+^, 755 [M+H-162-146]^+^, 593 [M+H-162-146-162]^+^, 431 [M+H-162-146-162-162]^+^, and *m/z:* 1061 [M-H]^−^, 899 [M-H-162]^−^, 753 [M-H-162-146]^−^; ^1^H-NMR (500 MHz, pyridine-d_5_) and ^13^C-NMR (125 MHz, pyridine-d_5_): see Table 1.

## 4. Conclusions

A new furostanol saponin 26-*O*-β-D-glucopyranosyl-(25*S*)-5α-furostane-20(22)-en-12-one-3*β*, 26-diol-3-*O*-*α*-L-rhamnopyranosyl-(1→2)-[*β*-D-glucopyranosyl-(1→4)]-*β*-D-galactopyranoside (**1**) along with two known saponins, chloromaloside A (**2**) and 25(*R, S*)-5α-spirostane-12-one-3-β-ol-3-*O*-*β*-D-xylopyranosyl(1→2)-[*β*-D-xylopyranosyl(1→3)]-glucopyranosyl-(1→4)-[*α*-L-rhamnopyrano-syl(1→2)]-*β*-D-galactopyranoside (**3**), were isolated from the Fruits of *T. terrestris*. Many pharmaceutical preparations with these saponins as the active compound have been commercially available. Examples of these are “tribusaponins” and “Xin-nao-shu-tong”, which have been used for the treatment of cardiovascular disease. This finding may provide a hint in the search for new and bioactive components from this plant. 

## Figures and Tables

**Table 1:** ^1^H- and ^13^C-NMR spectral data of compound **1** (recorded at 500/125 MHz in Pridine-d_5_; δ in ppm, *J* in Hz).

No.	δc	δ_H_ (*J*, Hz)	No.	δc	δ_H_ (*J*, Hz)
1	36.5	0.88, 1.57	C-3		
2	30.0	3.86 (m)	Gal-1	100.1	4.79(d,7.3)
3	76.8	3.81(m)	2	77.1	4.43
4	34.4	1.79, 1.99	3	76.6	4.16
5	44.6	0.90	4	81.4	4.52
6	28.8	1.09	5	75.4	3.95
7	31.5	1.80, 1.99	6	61.2	4. 15,4.65
8	34.3	1.55	Glu-1	107.3	5.06 (d,7.6)
9	55.7	0.50	2	75.7	4.22
10	36.5		3	79.0	4.05
11	38.3	1.15, 1.37	4	72.3	3.78
12	213.0	0.99, 1.58	5	78.7	3.82
13	55.7		6	63.2	4.08, 4.46
14	54.3	0.78	Rha-1	102.5	6.10 (s)
15	33.9	1.35, 1.98	2	72.5	3.85
16	83.1	4.43	3	72.9	4.18
17	56.4	2.33	4	74.3	4.15
18	14.3	0.66 (s)	5	69.6	3.89
19	12.0	0.78 (s)	6	18.8	4.24, 4.42
20	103.2		C-26		
21	11.8	1.62 (s)	Glu'-1′	105.3	4.70 (d, 7.6)
22	153.		2′	75.4	3.98
23	23.8	2.12	3′	78.8	4.25
24	31.5	1.38, 1.75	4′	71.9	4.18
25	33.9	1.90	5′	78.7	3.92
26	75.4	3.48(dd,7.5,9.5) 4.07 (m)	6′	63.0	4.36, 4.53
27	17.2	0.90 (d, 6.8)			
